# Trying to Put the Puzzle Together: Age and Performance Level Modulate the Neural Response to Increasing Task Load within Left Rostral Prefrontal Cortex

**DOI:** 10.1155/2015/415458

**Published:** 2015-10-08

**Authors:** Eva Bauer, Gebhard Sammer, Max Toepper

**Affiliations:** ^1^Cognitive Neuroscience at the Centre for Psychiatry, University of Giessen, Am Steg 24, 35385 Giessen, Germany; ^2^Department of Psychology, University of Giessen, Otto-Behaghel-Straße 10, 35394 Giessen, Germany; ^3^Bender Institute of Neuroimaging, University of Giessen, Otto-Behaghel-Straße 10H, 35394 Giessen, Germany; ^4^Evangelic Hospital Bielefeld (EvKB), Department of Psychiatry and Psychotherapy Bethel, Research Department, Remterweg 69-71, 33617 Bielefeld, Germany; ^5^Evangelic Hospital Bielefeld (EvKB), Department of Psychiatry and Psychotherapy Bethel, Department of Geriatric Psychiatry, Bethesdaweg 12, 33617 Bielefeld, Germany

## Abstract

Age-related working memory decline is associated with functional cerebral changes within prefrontal cortex (PFC). Kind and meaning of these changes are heavily discussed since they depend on performance level and task load. Hence, we investigated the effects of age, performance level, and load on spatial working memory retrieval-related brain activation in different subregions of the PFC. 19 younger (Y) and 21 older (O) adults who were further subdivided into high performers (HP) and low performers (LP) performed a modified version of the Corsi Block-Tapping test during fMRI. Brain data was analyzed by a 4 (groups: YHP, OHP, YLP, and OLP) × 3 (load levels: loads 4, 5, and 6) ANOVA. Results revealed significant group × load interaction effects within rostral dorsolateral and ventrolateral PFC. YHP showed a flexible neural upregulation with increasing load, whereas YLP reached a resource ceiling at a moderate load level. OHP showed a similar (though less intense) pattern as YHP and may have compensated age-effects at high task load. OLP showed neural inefficiency at low and no upregulation at higher load. Our findings highlight the relevance of age and performance level for load-dependent activation within rostral PFC. Results are discussed in the context of the compensation-related utilization of neural circuits hypothesis (CRUNCH) and functional PFC organization.

## 1. Introduction

Orientation and navigation in everyday life require a permanent adaptation of the spatial memory system. Spatial information has to be constantly integrated, maintained, updated, and recalled. The efficient control and coordination of these processes depend on effective spatial working memory operations which find their neural substrate in an anterior-posterior network of particularly prefrontal and parietal brain regions [1–3]. Damage to this network caused by stroke or neurodegeneration, for example, causes working memory deficits. With the establishment and advancement of neuroimaging techniques in the last decade, however, it could be shown that working memory performance declines across the life span even in the healthy brain [4] and that this age-related decline is associated with both structural [5–8] and functional [9–15] cerebral changes. On the functional level, age-related alterations are evident for most regions of the spatial working memory network. Nevertheless, the focus of research lies on the prefrontal cortex because it is common sense that this brain region plays the most prominent role for age-related working memory decline [16]. By contrast, the kind and meaning of activation changes within prefrontal cortex are heavily discussed. In fact, there are many studies reporting either decreased prefrontal cortex activation in older compared to younger adults (“underactivation”) as a sign of functional deficits [17, 18] or increased prefrontal activation (“overactivation”) being interpreted as neural inefficiency [19, 20], a reduction of regional specificity [20–22] or compensatory neural mechanism for age-associated deficits [12, 23–25]. Specifically, neural compensation was attributed to a more bilateral activation of the prefrontal cortex as proposed by the* hemispheric asymmetry reduction in old adults* (HAROLD) model [26]. The reasons for these partly inconsistent results are manifold and can be attributed to differences in study design and methodology. In fact, one of the most important mediating factors seems to be performance quality. Prefrontal overactivation or increased bilaterality in the presence of an age-related performance decline, for example, would argue for neural inefficiency, reduced regional specificity, or failed compensation, whereas overactivation or increased bilaterality at a steady performance level may be signs of successful compensation. By contrast, reduced prefrontal activation in older adults associated with lower performance accuracy was consistently interpreted as neural dysfunction [9, 10, 27–33].

*CRUNCH*. A second important mediating factor next to performance accuracy is the working memory load level of the applied paradigm. In fact, the* Compensation-Related Utilization of Neural Circuits Hypothesis* (CRUNCH) by Reuter-Lorenz and Cappell [34] proposes that the kind and meaning of activation differences between older and younger adults are strongly dependent not only on performance quality, but also on the cognitive demands of the applied task: older adults, in comparison to younger adults, show comparable performances at a low demand level but more intense or bilateral prefrontal activation indicating a recruitment of additional neural resources as compensatory response to limited working memory capacity. At high task demands, by contrast, older adults show poorer working memory performances accompanied by decreased prefrontal activation, pointing toward limited neural resources and failed compensation [10, 13, 27, 28, 32, 35].

*Prefrontal Cortex Organization.* Finally, the impact of performance level and task load on age-related changes in prefrontal brain activation might vary across specific subregions of the prefrontal cortex. For example, age-related compensatory overactivation could manifest in dorsolateral but not ventrolateral prefrontal areas. Consequently, other approaches refer to a functional prefrontal cortex organization and specific age-related changes within its subregions. Initially, dorsolateral prefrontal parts of the prefrontal cortex were attributed to higher-level cognitive processes, whereas the ventrolateral prefrontal cortex was rather related to the relatively passive maintenance of information [36–41]. Following this dorsolateral-ventrolateral distinction, Rypma and colleagues proposed that aging affects particularly dorsolateral parts of the prefrontal cortex (control processes), whereas the ventrolateral prefrontal cortex (maintenance) is relatively spared from age-related neural change [33, 42]. Later, Rajah and D'Esposito [22] adapted these assumptions by attributing bilateral ventrolateral prefrontal activation changes to the dedifferentiation of cortical function, right dorsolateral and anterior prefrontal activation changes to functional deficits, and left dorsolateral and anterior prefrontal cortex activation changes to functional compensation.

However, more recent research rather points toward a hierarchical rostral-caudal functional distinction of the prefrontal cortex with parallel dorsal and ventral processing streams [43, 44]. According to this theory, rostral parts of both dorsolateral and ventrolateral prefrontal cortices are associated with higher-level cognitive control, whereas caudal parts are rather linked to spatial maintenance [45, 46]. Age-related changes particularly seem to affect more rostral parts along this rostral-caudal gradient leading to reduced executive control [47–49].

Noteworthy, the assumptions of the described theories are not mutually exclusive and particularly highlight the relevance of rostral parts of the dorsolateral prefrontal cortex for top-down working memory control processes. In fact, recent research of our working group revealed a load × age interaction [47] and group differences between older high and low performers [50] within rostral dorsolateral prefrontal cortex, whereas there were hardly any effects within caudal or ventrolateral areas.

*Objectives*. Overall, the literature on this topic suggests that the kind and meaning of age-related prefrontal activation changes varies across prefrontal subregions and is highly dependent on performance level and task load. This implies the claim for further studies analyzing the impact of all of these factors with a single approach. Many past studies did not, which also applies to our preliminary work: the first one of the referred studies did not include comparisons between high and low performers while working memory load was not manipulated in the second one. In the current experiment, we therefore analyzed the effects of performance level, working memory load, and age by comparing load-dependent brain activation in younger high performers, younger low performers, older high performers, and older low performers. Functional magnetic resonance imaging (fMRI) was used to examine brain activation of different prefrontal subregions during working memory retrieval. Based on the theoretical considerations mentioned above, age-related differences in load-dependent brain activation should particularly manifest within rostral parts of the dorsolateral prefrontal cortex. Thereby, a successful recruitment of additional neural resources should be reflected by increasing activation with higher load and performance level, whereas an unsuccessful recruitment should be reflected by unchanged or even decreasing activation with lower load and performance level. In particular, a successful recruitment should be observed in younger high performers, whereas an unsuccessful recruitment should be most obvious in older low performers. Of particular interest is the comparison between older high performers and younger low performers: in fact, older high performers, unlike younger low performers, might show a similar neural response pattern as younger high performers reflecting an at least partly successful compensation of age-related behavioral working memory deficits [10, 51].

## 2. Materials and Methods

### 2.1. Participants

The study included a group of 19 younger participants and a group 21 older participants with normal or corrected-to-normal vision. To analyze the impact of performance level, both age groups were further subdivided into high performers and low performers by median split (errors in the experimental paradigm). Overall, four experimental groups were analyzed (Table 1): younger high performers (YHP), younger low performers (YLP), older high performers (OHP), and older low performers (OLP).

None of the participants had a documented diagnosis of neurological or psychiatric disease in the past. Moreover, global cognitive deficits were excluded by the Montreal Cognitive Assessment (MOCA) [52]. Participants were recruited by local advertising and provided a written declaration of consent prior to study start. The study obtained ethical approval by the Institutional Review Board of the University of Giessen. All participants received an expense allowance of 8 € per hour.

YHP and YLP did not differ with respect to age, gender, school education, and MOCA score, neither did OHP and OLP. Noteworthy, YHP and OHP differed with respect to years of school education (*t*(17) = 2.64; *p* = 0.017). However, due to differences between today's general school system and former systems, the average time of received school education in years is not really comparable between younger and older participants. Consequently, the multiple choice vocabulary test (MWT) [53] was additionally applied to test for possible age-related intellectual and educational differences. The MWT is a valid German questionnaire to estimate crystallized intelligence. Its total score is a predictor for the level of education. The four experimental groups did not differ with respect to MWT scores.

### 2.2. Task and Experimental Procedure

**Figure 1:**
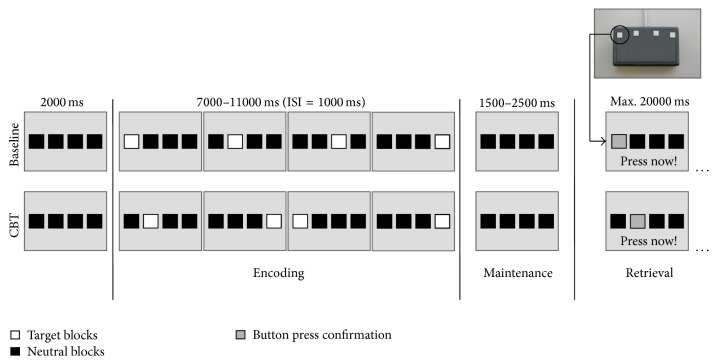
Exemplary illustration of the experimental design for load level 4. CBT = Corsi Block-Tapping test, ISI = interstimulus interval.

To assess spatial working memory, a modified electronic version of the Corsi Block-Tapping test (CBT) [47, 54] was applied. The CBT is a multiple-item spatial working memory task requiring the storage and reproduction of spatial target sequences. It allows modulating working memory load by variation of sequence length. The modified version provides four potential target locations (instead of nine as in the original version) indicated by four horizontally arranged blocks (Figure 1). Locations are randomly presented one after another and have to be reproduced in the correct temporal order afterwards. The original [55, 56] and the modified [47] versions of the CBT were associated with nearly identical whole-brain activation patterns indicating that the same cognitive and neural processes are involved.

Participants were instructed to learn (encoding phase), maintain (maintenance phase), and reproduce (retrieval phase) sequences of randomly presented target locations. Sequence length was varied between four (load 4), five (load 5), and six (load 6) locations in a row. In the baseline condition, all four target locations were presented from left to right. The chronological order of the different experimental conditions (baseline, load 4, load 5, and load 6) was pseudorandomized but equal for all participants. Participants were instructed to reproduce the sequence by sequential button presses after the presentation of each sequence. Therefore, a keypad with four horizontally arranged buttons was designed. Each of these four buttons represented the corresponding block on the screen. As direct feedback for the participants, each button press was confirmed by a change of the respective block's color.

Each trial of the CBT can be subdivided into an encoding phase (stimulus presentation), a maintenance phase (delay period), and a retrieval phase (stimulus reproduction). The encoding phase was preceded by a pause of 2000 ms. The encoding phase started with the onset of the first target block of every sequence and ended after the presentation of the last target block of that sequence. Duration of the target blocks was 1000 ms with a 1000 ms interstimulus interval. Due to different load levels (4, 5, and 6), the length of the encoding phase varied between 7000, 9000, and 11.000 ms. Each encoding phase was followed by a maintenance phase varying between 1500 and 2000 ms (variable jitter) [57] in which only the four horizontal blocks were shown. After the maintenance phase, the retrieval phase started indicated by the instruction “Press now” at the bottom of the screen. The retrieval phase lasted until the time of the final response. Maximum available time for making responses was set to 20.000 ms. The length of this time period was determined based on the results of preliminary studies (e.g., [47]).

Participants had to perform four trials per CBT sequence length as well as eight baseline trials. Consequently, 20 trials were randomly administered. Total duration of the experiment was about 10 minutes. Before entering the MRI examination room, participants obtained precise instructions concerning the experimental procedure. Subjects were instructed to memorize the correct locations and temporal order of the presented target blocks. For retrieval, participants were advised to reproduce the presented target sequences by successive button presses and to respond as fast and as accurate as possible. In addition, subjects had to perform a series of practice trials on a PC outside the scanner. Practice trials included two baseline trials and one load 5 trial. Duration of the practice session was about 2 minutes.

### 2.3. Stimulus Material

In the modified version of the CBT, four horizontally arranged black blocks (RGB 0 0 0) were displayed on gray background (RGB 163 163 163). Target blocks were displayed in red (RGB 255 0 0). In the retrieval phase, the black blocks turned to yellow (RGB 255 255 0) at button press to indicate the given response.

### 2.4. Data Acquisition

Functional and structural images were acquired using a 3 Tesla Siemens Magnetom Verio Scanner. Functional images were obtained using a T2^*∗*^-weighted echo planar imaging (EPI) sequence. Each volume contained 30 slices covering the whole brain, measured in descending order parallel to the AC-PC line + 25° (slice thickness = 4 mm; 1 mm gap; TR = 2100 ms; TE = 30 ms; flip angle = 90°; field of view = 192 × 192 mm; matrix size = 64 × 64; voxel size = 3 × 3 × 4 mm). Visual stimuli were displayed on a screen near the tube end, which participants saw via a dual-mirror mounted to the head coil. To control for inhomogeneity of the magnetic field, field map sequences were realized before the EPI sequence. Structural image acquisition consisted of 160 T1-weighted sagittal images with 1 mm slice thickness using a magnetization prepared rapid gradient echo (MPRage) sequence. Time of acquisition in the scanner was approximately 20 minutes per individual.

### 2.5. Data Analysis

#### 2.5.1. Behavioral Data Analysis

Behavioral data analysis comprised a 3 (CBT condition: load 4, load 5, and load 6) × 4 (group: YHP, YLP, OHP, and OLP) repeated measure ANOVA for the number of CBT errors. Bonferroni-tests were used for post hoc comparisons. Demographic group differences of interest (age, gender, education, and MoCA) were analyzed using two-sample *t*-tests and Chi-square tests, respectively. Behavioral data were analyzed using SPSS Statistics 22. All levels of significance were *α* = 0.05 and two-tailed.

#### 2.5.2. Brain Data Analysis

FMRI data were analyzed using SPM8 (Statistical Parametric Mapping Software; Wellcome Institute of Neurology at University College, London, UK; http://www.fil.ion.ucl.ac.uk/spm/). The first three images of every EPI-recording session were discarded to account for the time needed for the magnetic field to achieve a steady state. Preprocessing of EPI-images included unwarping and realignment to the first volume (b-spline interpolation), slice time correction, normalization to the standard space of the Montreal Neurological Institute (MNI) brain, and smoothing with an isotropic three dimensional Gaussian kernel with a full-width-at-half-maximum (FWHM) of 9 mm. Data were analyzed using a general linear model (GLM) with four encoding regressors (load 4 encoding, load 5 encoding, load 6 encoding, and baseline encoding), one regressor for the maintenance phase, and four retrieval regressors (load 4 retrieval, load 5 retrieval, load 6 retrieval, and baseline retrieval) (We included all trials instead of only correct trials into brain data analyses. Although past work has shown that results do not differ very much, this point is often heavily discussed. In fact, all aging studies are confronted with this problem since both options include pros and cons: analyzing all trials leads to higher error variance, whereas analyzing only correct trials leads to a different number of analyzed trials in the different experimental groups. Particularly in experimental designs modulating the load level, analyzing only correct trials that might lead to statistical effects: in the current work, e.g., the number of correct trials decreased with increasing load but this load-related decrease differed between the different experimental groups. Consequently, group × load interaction effects may be the statistical consequence of different trial numbers and not the consequence of activation differences. To avoid this, we decided to include all trials into brain data analyses in the current work). Compared to a model with single regressors for the maintenance phase (i.e., load 4 maintenance, load 5 maintenance, load 6 maintenance, and baseline maintenance), the present design helped to minimize correlations between the regressors of interest and the other predictors of the model. Timing of regressors followed the timing as explained in section above. In addition, six movement regressors were included into the design. Regressors were convolved using the hemodynamic response function as provided in SPM8. Design matrix was high pass filtered (128s). Since the present study focused on age-related changes during spatial working memory retrieval, only the retrieval regressors (load minus baseline) were further analyzed on the second level. A 4 × 3 factorial design matrix with the factor group (YHP, YLP, OHP, and OLP) and the factor load (load 4, load 5, and load 6) was realized using a flexible factorial model. Analyses focused on load-dependent cerebral activation (main effect of load) as well as the impact of age and performance level on this activation pattern (group × load interaction). Brain activation was analyzed at whole-brain level and by a region of interest (ROI) approach. For exploratory whole-brain analyses, a threshold of *Z* ≥ 3.1 with a minimum cluster size of 10 voxels was used. Based on the theoretical considerations in the introduction section, ROI analyses comprised a priori chosen brain regions located in the prefrontal cortex: Brodmann area (BA) 10 within the anterior prefrontal cortex, BAs 9 and 46 within the dorsolateral prefrontal cortex, and BAs 44 and 45 within the ventrolateral prefrontal cortex. Data were analyzed using the corresponding ROI masks of the automated anatomical labelling atlas (AAL) [58] which is implemented in the WFU PickAtlas [59], an automated software toolbox for generating ROI masks based on the Talairach Daemon database [60–62]. All reported ROI results were tested at a local significance threshold of *p* < 0.05 (voxel level). Alpha adjustment for multiple comparisons was done for each ROI (family-wise error (FWE) correction). Bonferroni adjustments for the number of tested ROIs are optionally provided in the results section. To describe the significant group × load interaction in more detail, contrast values of the identified peak voxels were extracted for each group and load level separately (Figure 4). Using this approach, we were able to get an idea where systematic variance exceeded random variance for comparisons of special interest (*t*-tests uncorrected for multiple comparisons).

## 3. Results

### 3.1. Behavioral Data

**Figure 2:**
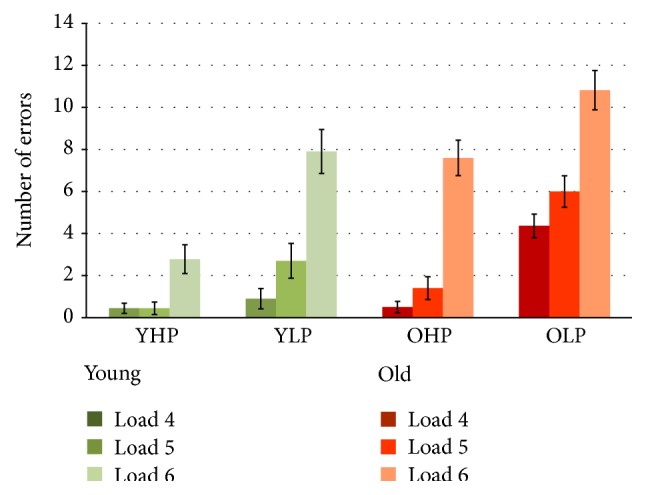
Number of CBT errors for each group and load level separately (displayed together with standard errors of the means). YHP = younger high performers; YLP = younger low performers; OHP = older high performers; OLP = older low performers.

For the number of errors (Figure 2), repeated measures ANOVA revealed significant main effects of group (*F*(3,36) = 29.28, *p* < 0.001) and load (*F*(2,72) = 89.88, *p* < 0.001) as well as a significant group × load interaction effect (*F*(6,72) = 3.25, *p* = 0.007). Post hoc Bonferroni-tests revealed that error rates of YLP and OHP did not differ. Besides, significant differences were found for all groups and load levels (*p* < 0.05). Results show that YHP made less errors than YLP, OHP, and OLP, with OLP showing the highest error rates. Moreover, error rates were higher at higher task load.

### 3.2. fMRI Data

**Figure 3:**
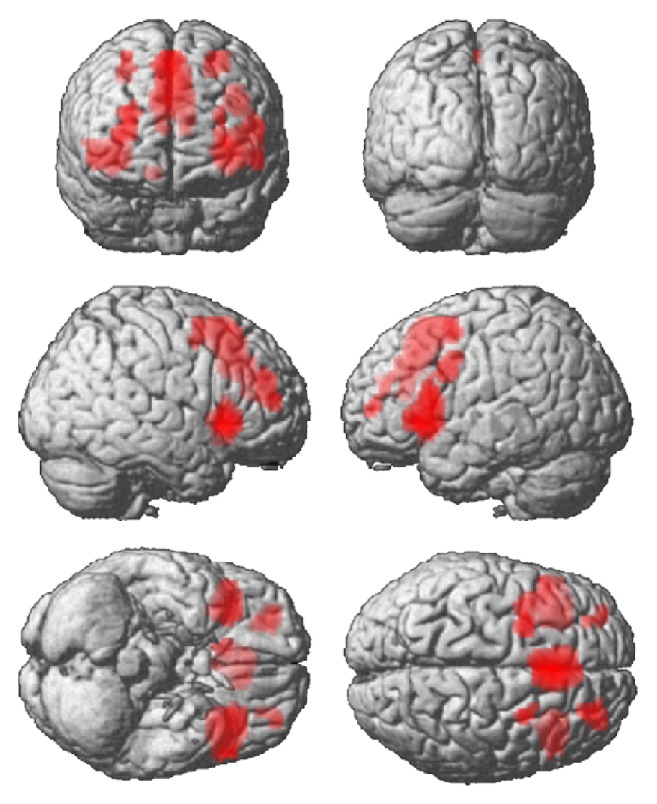
Main effect of load (whole-brain analysis with a threshold of *Z* ≥ 3.1).

Whole-brain analysis resulted in a significant main effect of load including different prefrontal brain regions (Figure 3, Table 2).

ROI analyses revealed increased load-dependent activation in bilateral dorsolateral (BAs 9 and 46), ventrolateral (BAs 44 and 45), and anterior prefrontal (BA 10) cortices (Table 3). Results indicate that the neural response pattern associated with task load includes various subregions of the prefrontal cortex.

For group × load interaction, whole-brain analysis revealed significant effects in the prefrontal cortex (Table 4).

ROI analyses confirmed significant interaction effects for left BAs 44 and 45 within the ventrolateral prefrontal cortex and for left BA 46 within the rostral dorsolateral prefrontal cortex (Table 5).

**Figure 4:**
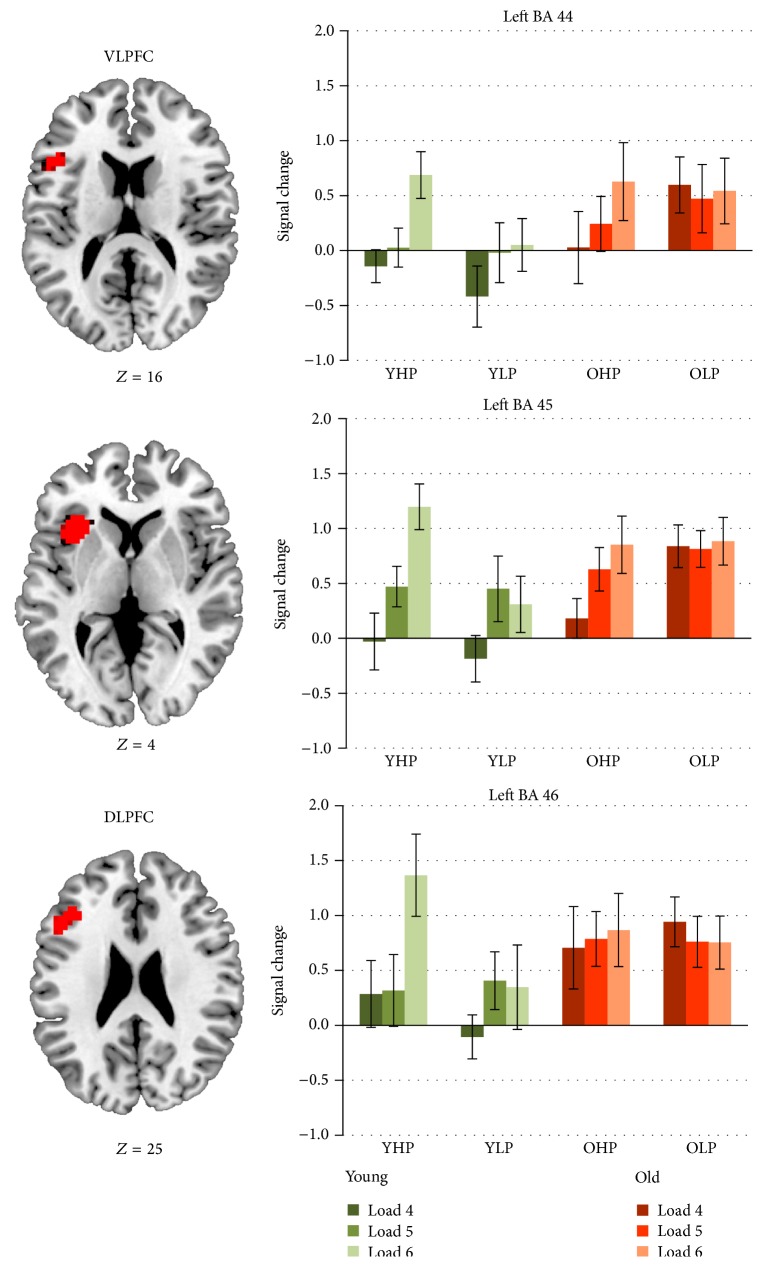
Contrast estimates with the respective standard errors for the identified regions associated with a group × load interaction. Signal change is plotted for younger high performers (YHP), younger low performers (YLP), older high performers (OHP), and older low performers (OLP) for the three load levels separately.

Signal changes in the respective peak-voxels are displayed in Figure 4 indicating comparable activation patterns in all prefrontal subregions. In particular, results show that systematic variance exceeded random variance for load 6 > load 4 in YHP within left BAs 44, 45, and 46 and in OHP within left BAs 44 and 45. OLP showed the opposite pattern in left BA 46 with higher activation intensity at load level 4 than at load level 6. In YLP, systematic variance exceeded random variance for load 5 > load 4 in left BAs 45 and 46 but not for load 6 > load 5. Group comparisons revealed that YHP showed less activation than OLP at load level 4 within left BAs 44 and 45 (same tendency in left BA 46), but more left-hemispheric BA 45 activation than YLP at load level 6 (same tendency in BAs 44, 46). Compared with OHP, OLP showed higher activation intensity at load level 4 within left BA 45. Finally, the bar charts suggest a tendency toward higher activation in OHP than in YLP at load level 6 within BAs 44, 45, and 46.

## 4. Discussion

In the current study, we used fMRI to investigate the effects of age, performance level, and load on prefrontal brain activation associated with spatial working memory retrieval. The results highlight the relevance of age and performance level for load-dependent activation within left rostral dorsolateral and ventrolateral prefrontal cortices. In line with the assumptions of the CRUNCH model [34], our results suggest that younger high-performing individuals show a flexible upregulation of activation as neural response to increasing task load, whereas younger low performers seem to reach a resource ceiling at a moderate load level. Older high performers show a similar though less intense pattern than younger high performers and may compensate age-effects at high task demands. By contrast, older low performers seem to show neural inefficiency at low task demands and no upregulation of the working memory network if task demands rise.

### 4.1. Spatial Working Memory Performance

In line with previous research [10, 47, 56], analyses of behavioral data revealed an increasing number of errors with load across all participants. The different experimental groups showed accuracy differences across all load levels with younger high performers showing the best and older low performers showing the poorest performances. Moreover, analyses revealed a significant group × load interaction indicating that the increase of task load differentially affected the increase of errors in the different experimental subgroups. Most interestingly, younger low performers and older high performers did not only show similar error rates across all load levels but also a similar increase of errors with increasing load. Latter findings highlight that higher age is not always associated with lower performance accuracy.

### 4.2. Effects of Age, Load, and Performance Accuracy on Prefrontal Cortex Activation

Brain data analyses identified a load-dependent frontal network across all participants. In particular, dorsolateral (BAs 9, 46), ventrolateral (BAs 44, 45), and anterior (BA 10) prefrontal cortices showed an upregulation associated with task load. These findings are in line with previous research and point toward the relevance of prefrontal brain structures for flexible working memory processes that control the adaptation of neural resources to the demands of the applied task [2].

Moreover, the study design allowed assumptions about the impact of age and performance level on the upregulation of this load-related network. Brain data analysis revealed significant group × load interaction effects within rostral parts of different left-hemispheric dorsolateral and ventrolateral prefrontal subregions. These interaction effects indicate that the neural response to task load differed between the four experimental groups. Results suggest that younger high performers showed a sharp increase of activation intensity from the lowest to the highest load level (left BAs 44, 45, and 46). Moreover, they showed higher activation intensity than younger low performers at high task demands (left BA 45; same tendency in BAs 44 and 46) but lower activation intensity than older low performers at low task demands (left BAs 44 and 45; same tendency in 46). Together, these findings confirm the assumptions of the CRUNCH model and suggest a flexible and effective recruitment of additional resources in younger high-performing individuals to meet the demands of higher task load [34]. By contrast, this neural response seems to be qualitatively different and less effective in younger and older low performers. Following this argumentation, younger low performers showed an upregulation from low to moderate load (left BAs 45 and 46) but no further increase at high task load proposing that a resource ceiling has been reached. Older low performers appeared to show steady (left BAs 44 and 45) or even reduced (left BA 46) activation intensity with increasing load suggesting that neural resources were already exhausted at the lowest load level.

In addition, the current findings illustrate that the CRUNCH effects are modulated by the performance level of younger and older individuals. In fact, an efficient upregulation of rostral prefrontal cortex activation as neural response to higher task load could not only be observed in* younger* high-performing individuals but, to lesser degree, in* older* high performers (left BAs 45 and 46). In fact, older high performers showed a similar though less intense pattern of upregulation from the lowest to the highest load level as younger high performers which most likely indicates a qualitatively similar neural response [10, 51]. In particular, the bar charts suggest a tendency toward higher activation intensity in older high performers compared to younger low performers at high task demands (left BAs 44, 45, and 46). In the context of equivalent error rates on the behavioural level, these findings point toward compensation of age-related deficits in older high performers. By contrast, older high performers showed lower activation intensity than older low performers at low task load (left BA 45) suggesting that high activation intensity at low task load rather reflects neural inefficiency than compensation. Together, the latter findings suggest that older high-performing individuals may show compensation at high task load and less neural inefficiency than their low-performing counterparts at low task load.

Overall, our findings indicate that age and performance level modulate the cerebral response to working memory load. Younger high performers and older high performers show a qualitatively similar flexible upregulation of prefrontal activation as neural response to increasing task load, whereas younger and older low performers show a different and less effective neural response because, sooner or later, resource ceilings are reached. Noteworthy, our findings are quite in line with the results of Nagel and colleagues [10]. In particular, the load-related activation patterns within left dorsolateral prefrontal cortex are amazingly similar confirming the validity of these effects. Moreover, the current results suggest that activation patterns are quite the same for different spatial working memory subprocesses since Nagel and colleagues verified these effects in a recognition task whereas our results refer to working memory retrieval. For retrieval, our results additionally suggest neural compensation in older high performers at high task load and neural inefficiency in older low performers at low task load. Finally, specific effects were not only found in dorsolateral but also in ventrolateral prefrontal subregions which provides important information about the functional organization of the prefrontal cortex.

### 4.3. Prefrontal Cortex Organization

As mentioned above, the prefrontal cortex can be subdivided into different functional modules. Whereas some authors propose a hierarchical dorsolateral-ventrolateral distinction with dorsolateral parts being related to higher-level working memory operations (e.g., control processes) and ventrolateral parts to passive maintenance [36–41], recent research suggests a rostral-caudal distinction with rostral parts reflecting working memory control and caudal parts being associated with maintaining information [43–46]. The results of the current work rather support the second idea, since the patterns of activation intensity at the different load levels differed between the experimental groups (interaction effect) but were, in each experimental group, quite the same for dorsolateral and ventrolateral prefrontal subregions. These findings suggest that functional differences between dorsolateral and ventrolateral prefrontal cortices may be less evident than we thought. Instead, interaction effects were located within more rostral parts of both regions (i.e., BA 46, BA 45, and anterior BA 44) indicating that particularly* rostral* parts of the prefrontal cortex are associated with age, performance level, and task load. Finally, overall activation was more intense in rostral than in caudal areas (i.e., higher activation intensity in BAs 46 and 45 than in BA 44 and no differences between BAs 46 and 45). Taken together, our findings point toward a hierarchical rostral-caudal prefrontal cortex organization and suggest that age-related alterations modulated by performance level and task load particularly manifest within different rostral regions along this axis.

### 4.4. Limitations and Perspectives

Noteworthy, the interpretation of regional activation intensity at the different load levels relies on descriptive results. However, the interaction effects prove that the neural response to task load differs between the four experimental groups and the post hoc comparisons verify most of the differences the bar charts suggest. Moreover, there are very similar load-related activation patterns in different prefrontal subregions (left BAs 44, 45, and 46). These patterns are fairly identical with the activation patterns identified by Nagel and colleagues which further increases the validity of the data. The strength of the current work certainly is that age, performance level, and task load are included into one analysis. Finally, the results show that, dependent on performance level and task load, overactivation may reflect either neural inefficiency or compensation. Future research should address a further distinction between these processes if possible. In addition, future studies should focus on the question how the neural response to increasing task load is affected by neurodegenerative disorders. In fact, there is evidence that increased task demands provoke a disproportionate performance decline in patients suffering from Alzheimer's disease and mild cognitive impairment [63, 64].

## Figures and Tables

**Table 1:** Sample characteristics.

	YHP	YLP	OHP	OLP
*N*	9	10	10	11
Gender (female/male)	4/5	7/3	5/5	6/5

Mean age/SD	27.89/5.18	27.3/5.33	59.5/5.46	61.91/5.03
Minimum age	20	21	50	56
Maximum age	35	35	68	71

School education/SD	12.67/1.0	12.4/1.08	11.0/1.63	11.09/1.92
Minimum school education	10	10	8	8
Maximum school education	13	13	13	13

MoCA score/SD	29.11/.33	27.2/2.74	27.1/2.73	26.0/2.61

MWT score/SC	32.44/2.7	30.6/3.41	32.3/3.56	31.73/3.95

*Note*. Age and school education are given in years. YHP = younger high performers; YLP = younger low performers; OHP = older high performers; OLP = older low performers; SD = standard deviation; MoCA = Montreal Cognitive Assessment; MWT = multiple choice vocabulary test.

**Table 2:** Localization and statistics of the peak voxels for the main effect of load (whole-brain analysis).

Brain structure	Cluster size	*x*	*y*	*z*	*F*
L inferior frontal gyrus, triangular part/insula	384	−33	20	4	23.16
L superior frontal gyrus, medial part	527	−3	23	43	22.62
R insula	207	36	20	−2	21.05
L middle frontal gyrus	54	−24	5	55	12.10
L precentral gyrus	69	−39	2	34	11.21
R middle frontal gyrus	94	27	44	16	10.12
R middle frontal gyrus	32	27	5	58	9.34
L middle frontal gyrus	26	−33	53	7	9.17

*Note*. Threshold of *Z* ≥ 3.1. All coordinates (*x*, *y*, *z*) are given in MNI space. L = left; R = right.

**Table 3:** Localization and statistics of the peak voxels for the main effect of load (ROI analyses).

PFC subregion	ROI	Brain structure	*x*	*y*	*z*	*F*	*p*_corr_
DLPFC	BA 9	L superior frontal medial gyrus	−6	29	37	12.35	0.003^*∗*^
		R cingulum middle	3	32	34	14.18	0.001^*∗*^
	BA 46	R inferior frontal, opercular part	48	17	28	8.27	0.026
VLPFC	BA 44	L inferior frontal, opercular part	−51	17	16	13.99	>0.001^*∗*^
		R inferior frontal, opercular part	51	17	7	7.17	0.028
	BA 45	L insula	−36	23	4	19.42	>0.001^*∗*^
		R insula	39	23	4	11.42	0.002^*∗*^
aPFC	BA 10	R middle frontal gyrus	27	44	25	9.89	0.022

*Note*. Threshold of *p*_corr_ < 0.05 (FWE-corrected according to SPM8, small volume correction). *∗* indicates results surviving a Bonferroni-correction for the set of ROIs (*p*_corr_ < 0.005). All coordinates (*x*, *y*, *z*) are given in MNI space. DLPFC = dorsolateral prefrontal cortex; VLPFC = ventrolateral prefrontal cortex; aPFC = anterior prefrontal cortex; ROI = region of interest; BA = Brodmann area; L = left; R = right.

**Table 4:** Localization and statistics of the peak voxels for the load × group interaction (whole-brain analysis).

Brain structure	Cluster size	*x*	*y*	*z*	*F*
L middle frontal gyrus	149	−27	−1	52	7.90
L insula	74	−36	17	4	6.71
R superior frontal gyrus	55	27	2	61	6.10
L supplementary motor area	80	0	23	46	5.85
R insula	23	33	23	−5	5.78
L inferior frontal gyrus, triangular part	16	−48	20	22	5.04

*Note*. Threshold of *Z* ≥ 3.1. All coordinates (*x*, *y*, *z*) are given in MNI space. L = left; R = right.

**Table 5:** Localization and statistics of the peak voxels for the group × load interaction (ROI analyses).

PFC subregion	ROI	Brain structure	*x*	*y*	*z*	*F*	*p*_corr_
DLPFC	BA 46	L frontal inferior gyrus, triangular part	−51	23	25	4.48	0.030
VLPFC	BA 44	L frontal inferior gyrus, opercular part	−51	17	16	3.91	0.039
	BA 45	L insula	−39	20	4	5.10	0.007^*∗*^

*Note*. Threshold of *p*_corr_ < 0.05 (FWE-corrected according to SPM8, small volume correction). *∗* indicates results surviving a Bonferroni-correction for the set of ROIs (*p*_corr_ < 0.005). All coordinates (*x*, *y*, *z*) are given in MNI space. PFC = prefrontal cortex; DLPFC = dorsolateral prefrontal cortex; VLPFC = ventrolateral prefrontal cortex; ROI = region of interest; BA = Brodmann area; L = left; R = right.
